# Pivotal role of micro-CT technology in setting up an optimized lung fibrosis mouse model for drug screening

**DOI:** 10.1371/journal.pone.0270005

**Published:** 2022-06-15

**Authors:** Zahra Khalajzeyqami, Andrea Grandi, Erica Ferrini, Francesca Ravanetti, Ludovica Leo, Martina Mambrini, Luciana Giardino, Gino Villetti, Franco Fabio Stellari

**Affiliations:** 1 Department of Veterinary Medical Sciences, University of Bologna, Bologna, Italy; 2 Pharmacology & Toxicology Department, Chiesi Farmaceutici S.p.A., Corporate Pre-ClinicalR&D, Parma, Italy; 3 Department of Veterinary Science, University of Parma, Parma, Italy; 4 Department of Medicine and Surgery, University of Parma, Parma, Italy; Institut de Pharmacologie Moleculaire et Cellulaire, FRANCE

## Abstract

Idiopathic pulmonary fibrosis (IPF) is a progressive disease with no curative pharmacological treatment. The most used animal model of IPF for anti-fibrotic drug screening is bleomycin (BLM)-induced lung fibrosis. However, several issues have been reported: the balance among disease resolution, an appropriate time window for therapeutic intervention and animal welfare remain critical aspects yet to be fully elucidated. In this study, C57Bl/6 male mice were treated with BLM via oropharyngeal aspiration (OA) following either double or triple administration. The fibrosis progression was longitudinally assessed by micro-CT every 7 days for 4 weeks after BLM administration. Quantitative micro-CT measurements highlighted that triple BLM administration was the ideal dose regimen to provoke sustained lung fibrosis up to 28 days. These results were corroborated with lung histology and Bronchoalveolar Lavage Fluid cells. We have developed a mouse model with prolonged lung fibrosis enabling three weeks of a curative therapeutic window for the screening of putative anti-fibrotic drugs. Moreover, we have demonstrated the pivotal role of longitudinal micro-CT imaging in reducing the number of animals required per experiment in which each animal can be its own control. This approach permits a valuable decrease in costs and time to develop disease animal models.

## Introduction

Idiopathic pulmonary fibrosis (IPF) is a chronic lung disease for which no curative pharmacological therapy exists, which is characterized by progressively impaired pulmonary function and a median survival of 2–4 years after diagnosis [1].

Animal models play an essential role in selecting the most promising drug candidates to advance into the clinic. However, a very limited number of such compounds have translated into effective therapies to date, with many having failed to reach the primary endpoints in clinical trials [2, 3].

This unsuccessful translation of early promise into clinical benefit for IPF patients is a waste of financial resources, ultimately reducing the opportunity for conducting future trials.

A possible explanation could be related to the nature of currently available animal models, which cannot fully replicate human pulmonary fibrotic disease [4].

Although bleomycin (BLM)-induced fibrosis is the most used and best-characterized model either to investigate lung fibrotic mechanisms or to screen drugs, a comprehensive guideline reporting optimal doses, routes and frequency of BLM administration is still lacking.

The BLM model does not fully reproduce human IPF, and its limitations are well-known.

First, it is challenging to balance the development of a sustained fibrosis with current standards for animal welfare practices, as animals tend to lose weight and show signs of distress [4, 5]. Moreover, the spontaneous resolution of the resultant disease 21 days after its induction could be considered an obstacle to identifying an appropriate time window for pharmacological intervention, as observed in our female model [6].

Several studies have pointed out the different responses to BLM among different mice strains and genders [4]; as concerns C57Bl/6 strain, male mice have been reported to be more susceptible to bleomycin than females, namely due to a lower expression of the enzyme Bleomycin hydrolase [4, 7–9]. However, the precise mechanisms underlying either strain or gender specificity remain elusive.

Hence, the American Thoracic Society (ATS) and National Institute of Health (NIH) recommended using both genders for lung anti-fibrotic drugs screening since they respond differently to BLM [4, 7–9].

We have already set up our drug screening in the female model of BLM with two weeks for pharmacological intervention [6].

Following ATS and NIH suggestions, we intended to develop a mouse model of BLM-induced lung fibrosis in male, however, extending the window for therapeutic intervention up to three weeks and performing the primary screening in both genders.

In light of the different sensitivity among genders we applied lower BLM dosages than we did in females [6, 10], exploring different BLM administration regimens; on one hand, we examined the double oropharyngeal aspiration (OA), as we did in female mice, while on the other we tried to split the BLM into three doses.

Our main objective was to identify the ideal BLM dose regimen to reach an appropriate balance between sustained lung fibrosis up to 28 days and animal welfare in C57Bl/6 male mice, highlighting the pivotal role of micro-CT in animal modeling.

The extended therapeutic window to 3 weeks allows to reveal side effects due to longer drug exposition, as well as lighting up more in-depth the anti-fibrotic efficacy already in primary screening.

Micro-CT has been well-documented to play an important role in decreasing intra-experimental variability, and, as a result, significantly reducing the number of animals used per experiment, in agreement with the 3R’s principles [11]. In particular, micro-CT technology has been used to quantify lung fibrosis progression, as well as the evaluation of the response to pharmacological treatments [6, 11–13].

In this study, longitudinal micro-CT imaging was performed on the same mice at 7, 14, 21 and 28 days to assess the pulmonary fibrosis progression. Five quantitative parameters (Normo-, Hypo- and Non-Aerated tissue, Air/Tissue and FRC/V_exp_) were derived from CT scans and compared with histological measurements at the final time-point.

Refinement of the BLM model, as well as the integration of in-vivo imaging techniques, might help the drug discovery process to increase the reliability of preclinical data and the transferability of drug candidates to the clinic [14, 15].

## Material and methods

### Experimental animals

All studies were conducted in 7-8-week-old male C57Bl/6 mice (purchased from *Envigo*, *San Pietro al Natisone*, *Udine*, *Italy*). Animals were housed five per cage under standard conditions at our animal facility, in compliance with the procedures and principles outlined in the European Directive 2010/ 63 UE, Italian D.Lgs 26/2014 and the revised “Guide for the Care and Use of Laboratory Animals” (National Research Council Committee, US, 2011). All animal procedures were conducted in an AAALAC (Association for Assessment and Accreditation for Laboratory Animal Care) certified facility at Chiesi Farmaceutici and were authorized by the Italian Ministry of Health with protocol number 841/2019-PR and by the internal AWB (Animal Welfare Body). Animals were acclimatized upon arrival to the local vivarium conditions (room temperature: 20–24°C; relative humidity: 40–70%; 12-h light-dark cycle; food and water ad libitum) for 7–10 days. All appropriate measures were taken to minimize pain or discomfort in the animals; the pain was evaluated daily through a Visual Analogue Scale (VAS) ranging from 0 to 10 by a designated veterinarian or trained technicians. Signs of dyspnoea, body weight loss ≥ 20% and VAS ≥6 were considered as humane endpoints.

### Fibrosis induction

**Fig 1:**
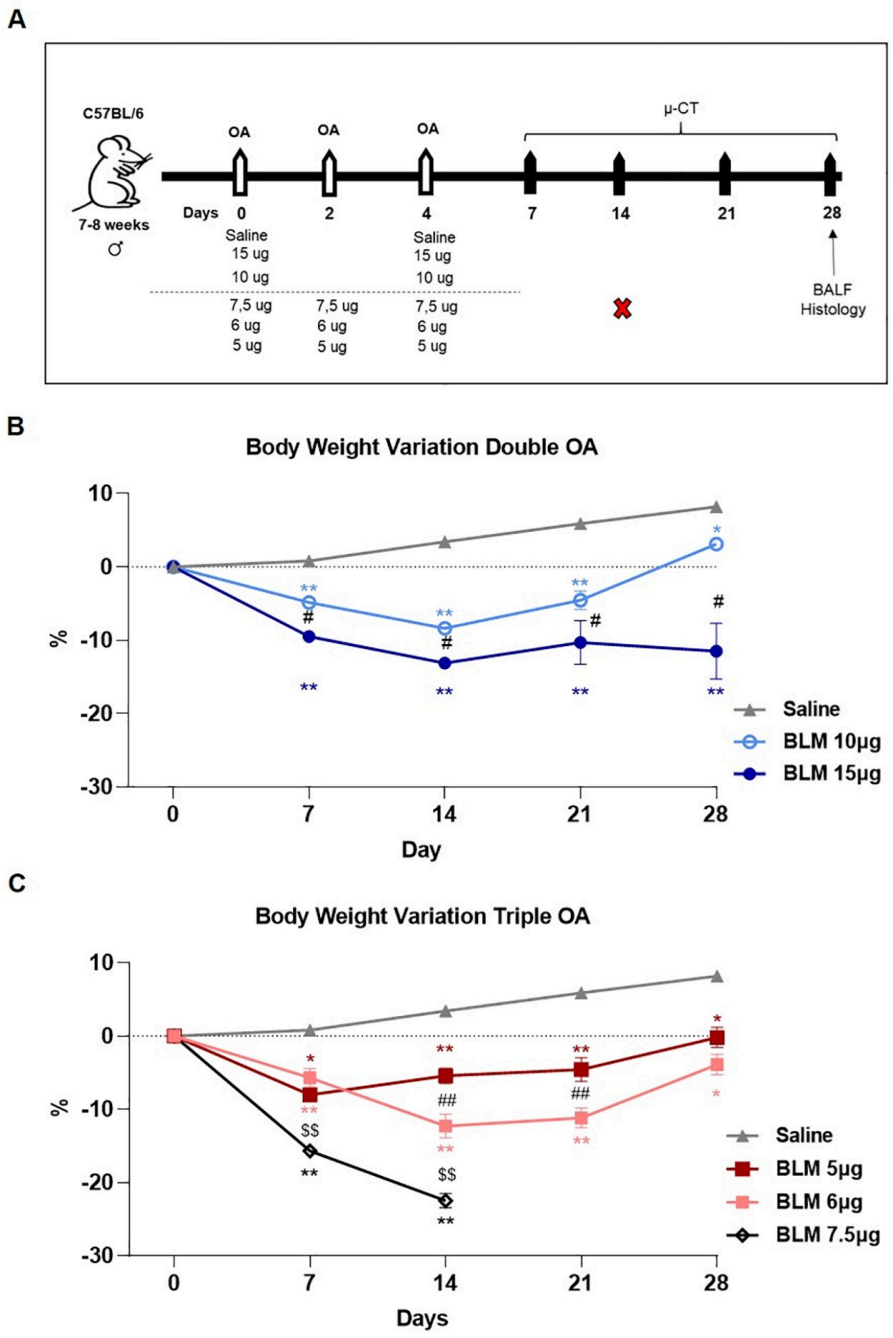
Schematic representation of the experimental setting and body weight variation. (A) Thirty C57Bl/6 male mice were randomized in 6 groups to receive saline, double oropharyngeal administration (day 0 and 4) of 10 or 15 μg of BLM, and triple OA (day 0, 2, 4) of 5, 6 or 7.5 μg of BLM. Micro-CT (μ-CT) was performed at days 7, 14, 21 and 28. Animals were euthanized at day 28 to collect BALF and lungs for histology. Body weight was reported as percentage of variation compared to the baseline (day 0) for saline, double OA (B) and triple OA (C) groups. Data were shown as mean ± SEM. Statistical analysis was performed via two-way ANOVA followed by Tukey’s test * p<0.05, ** p<0.01 vs. saline; # p<0.05, ## p<0.01 comparing 10 μg vs. 15 μg BLM and 5 μg vs. 6 μg BLM; $$ p<0.01 comparing 7.5 μg vs. 6 μg BLM). N = 5 per group.

Pulmonary fibrosis was induced using bleomycin hydrochloride (Baxter) diluted in 50 μL saline via a double (day 0, 4) or triple (day 0, 2, 4) oropharyngeal administration (OA) under 2.5% isoflurane anaesthesia, as previously described [6, 10, 11]. Briefly, male mice of 25±1 g were placed on an intubation stand and the liquid was drained into the distal part of the oropharynx with a micropipette. 30 mice (5 per group) were randomized to receive double administration with 10 or 15 μg BLM at each shot corresponding to (0.4 and 0.6 mg/kg), whereas those receiving triple administration were given 5, 6 or 7.5 μg BLM at each OA corresponding to (0.2, 0.25 and 0.3 mg/kg). The vehicle group received 50 μL saline at each administration. A schematic representation of the experimental procedure is shown in Fig 1A.

### Micro-computed tomography

Animals’ lungs were scanned longitudinally at days 7, 14, 21 and 28 by Quantum GX Micro-CT (PerkinElmer, Inc. Waltham, MA). Each mouse was anesthetized with 2% isoflurane and positioned supine inside the micro-CT. Images were acquired with the following parameters: 90KV, 88μA over a total angle of 360° in 4-minute-total scan time using the ‘high speed’ scan mode with respiratory gating. The 3D reconstructed datasets in the expiration phase of the breathing cycle were analysed by the Perkin Elmer Analyze software (Analyze 12.0; Copyright 1986–2017, Biomedical Imaging Resource, Mayo Clinic, Rochester, MN). A semi-automatic segmentation was used to define airways and aerated lungs, whereas manual segmentation was performed in animals with very low- or non-aerated areas to obtain the total lung volume map [11]. The images were rescaled from grey levels to Hounsfield Units (HU) through the linear transformation model (-1000 HU as the density of air and 0 HU as the density of water). Pre-clinical HU density ranges were applied to the lungs map to divide the parenchyma into normo-aerated [-860 HU; -435 HU], hypo-aerated [-435 HU; -121 HU] and non-aerated regions [-121 HU; 121 HU] [11]. The volume of each compartment was normalized and expressed in the percentage of total lung volume for all CT scans. Mean Lung Attenuation (MLA) was also extracted from each rescaled HU image at the end of the expiratory phase to quantify the content of air (also known as Functional Residual Capacity (FRC) [16, 17] and tissue in the lung, following the equations:

$$Air_{exp}\;or\;FRC\;\left(mm^{3}\right)=\;V_{exp}\left(mm^{3}\right)\frac{MLA_{exp}\left(HU\right)}{Air\;\left(HU\right)};$$ (1)

$$Tissue_{exp}\left(mm^{3}\right)=\;V_{exp}\left(mm^{3}\right)-\;Air_{exp}\left(mm^{3}\right);$$ (2)

FRC was then normalized on total lung volume and expressed as FRC/V_exp_.

Another functional CT parameter, i.e. air/tissue ratio (A/T), was derived and measured as follow:

$$A/T=\;\frac{Air_{exp}\left(mm^{3}\right)}{Tissue_{exp}\left(mm^{3}\right)};$$ (3)

According to this definition, dense and scarce aerated lungs are associated with a lower gas/tissue ratio, thus this parameter can be used to quantify progression of lung fibrosis.

### Bronchoalveolar lavage and cell count

Mice were sacrificed by anaesthetic overdose followed by abdominal aortic bleeding. Bronchoalveolar lavage fluid (BALF) was collected by gently washing the lungs three times with 0.6 mL sterile solution [Hank’s balanced salt solution (HBSS) 1x; ethylenediaminetetraacetic acid (EDTA) 10 mM; 4-(2-hydroxy-ethyl)-1-piperazineethansulphonic acid (HEPES) 10 mM]. The BALF samples were centrifuged for 10 mins at 4°C, and the cell pellet was resuspended in 0.2 mL of BAL solution to count white blood cells (WBC) and relative subpopulations with an automated cell counter (*Dasit XT* 1800J, Sysmex).

### Histology

Lungs were harvested after gentle infusion of 10% neutral-buffered formalin, fixed for 24 hours and processed for embedding. Sections of 5 μm thick were cut (Slee Cut 6062; Slee Medical, Mainz, Germany) and three sections for each lung sample were stained with Masson’s Trichrome (MT) and scored on a scale of 0 to 8. The fibrosis alterations were blindly assessed on Ashcroft score modified by Hubner et al. by two independent researchers with experience in animal models of lung fibrosis [18, 19]. The final score was expressed as a mean of individual scores observed across all microscopic fields. To quantify the distribution of pulmonary fibrosis, the Ashcroft scores were graded in three classes of increasing values ranging from 0 to 3 (mild), 4 (moderate), and ≥5 (severe). The whole slide images (WSI) were acquired by NanoZoomer S60 scanner (Hamamatsu Photonics, K.K., Japan).

### Statistical analysis

Statistical analyses were performed using Prism 8 software (*GraphPad Software Inc*., *San Diego*, *CA*, *United States*). All data are presented as mean ± SEM. One or two-way analysis of variance (ANOVA) was performed, followed by Dunnett or Tukey’s multiple comparison *post-hoc* tests to compare different experimental groups. Normal distribution was assessed through a Shapiro-Wilk test accompanied by a visual inspection of QQ-plots. The sample size was calculated with A-priori Power Analysis (G*Power Version 3.1.2) considering Ashcroft Score as an endpoint. For all the applied tests, a p-value <0.05 (*) was considered as statistically significant. Spearman correlation analysis was used to estimate the relation between the micro-CT parameters and Ashcroft score.

## Results

### Clinical observations

Experimental set up reporting the BLM doses, scheme of administration, time-points of observation is shown in Fig 1A.

BLM administration caused a dose-dependent body weight loss. The reduction of body weight was within 10% between day 0 and 14 in mice treated with either double (Fig 1B) or triple OA (Fig 1C), except for those receiving a triple 7.5 μg BLM dose, which showed a marked decrease in body weight in the first week, reaching 20% at 14 days. Thus, in agreement with the designated veterinarian and the internal Animal Welfare Body, this group of mice was sacrificed and excluded from the study (data collected until 14 days were reported in S1C–S1E Fig). All the mice receiving either BLM double or triple OA showed a recovery in body weight at later time-points, regaining the initial body weight at day 28, except for the group receiving double dose of 15 μg whose body weight remained unaltered from day 14 to day 28. Among these groups, no mortality was observed. Control mice treated with saline gained weight, as expected, during the time of observation.

### Micro-CT analysis

#### Lung aeration degrees

All mice were imaged longitudinally at 7, 14, 21 and 28 days.

**Fig 2:**
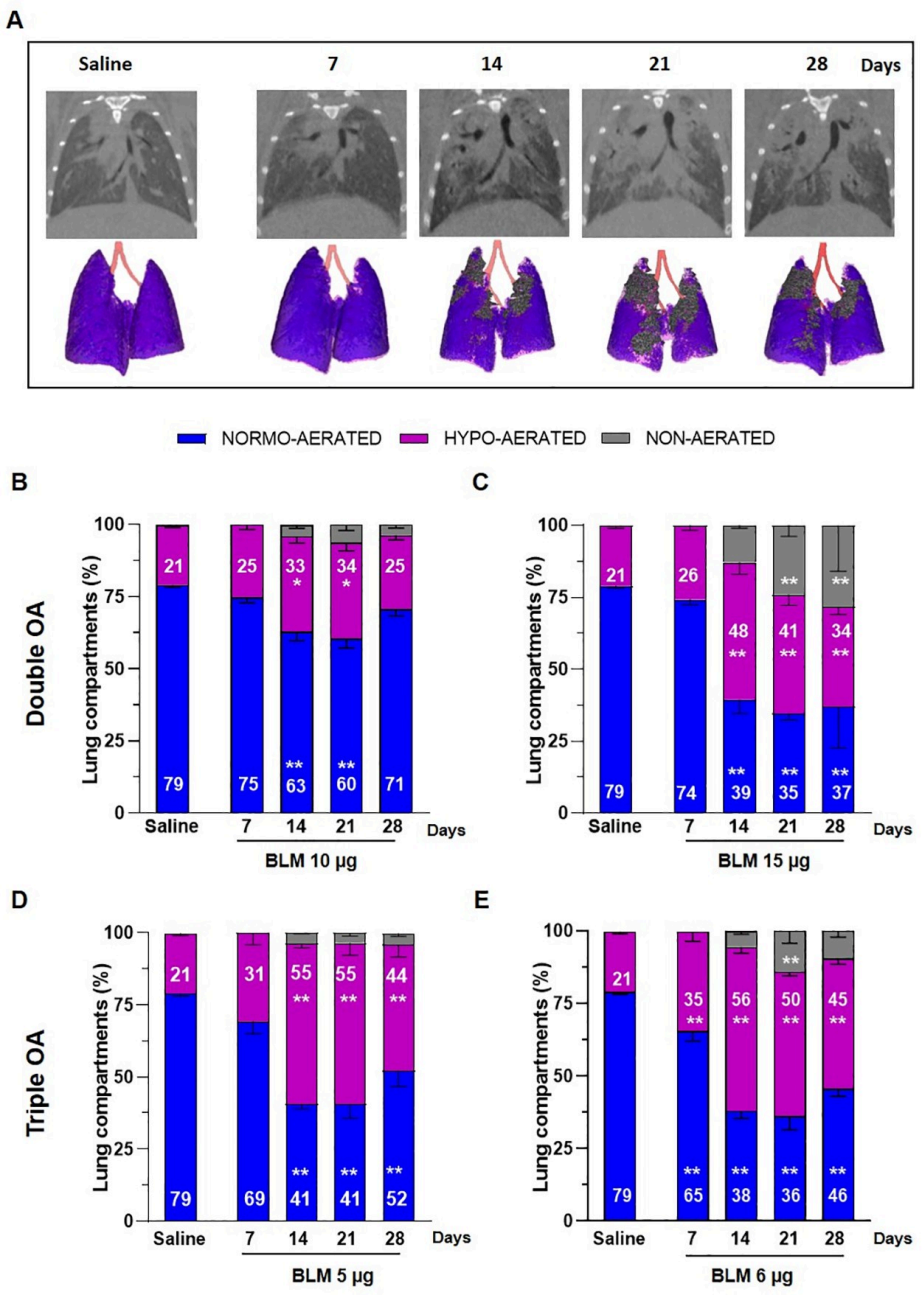
Longitudinal micro-CT assessment. (A) Representative coronal micro-CT lung slices and 3D renderings at the end of expiration phase of saline- and BLM-treated mice at 7, 14, 21 and 28 days. (B-E) Lung aeration degrees expressed as percentage of normo-, hypo-, and non-aerated tissues detected at 7, 14, 21, 28 days for the saline group and BLM groups at different dose regimens. Data are shown as mean ± SEM. The statistical analysis was performed for each group compared to saline via two-way ANOVA followed by Dunnett’s test (*p<0.05; **p<0.01 vs. saline). N = 5 per group.

Representative coronal micro-CT lung slices and the corresponding 3D renderings of BLM and saline-treated animals at different time-points are reported in Fig 2A. Lung 3D images showed hypo- and non-aerated areas in BLM mice at different time-points (Fig 2A), whereas representative images of saline-treated lungs mainly composed of normal lung parenchyma throughout of experiment were reported in S1A Fig. Animals treated with a double OA of 10 μg BLM showed a significant increase (p<0.05) in hypo-aerated tissue compared to saline at 14 and 21 days, which was no longer observed at day 28. Non-aerated lung tissue, corresponding to more severely damaged areas, was between 4–6% of the whole parenchyma since day 14, and remained stable up to the end of the study, not being significantly higher than in the saline group (Fig 2B).

The double administration of 15 μg BLM caused a marked increase (p<0.01) of hypo-aerated tissue from day 14 to day 28, followed by a significant augment of non-aerated areas (p<0.01) at day 21 and 28 (Fig 2C). As regards mice treated with the triple dose regimen, those receiving 5 μg BLM showed an increased hypo-aerated tissue between days 14–28 (p<0.01), whilst non-aerated areas remained at about 4% of the lung parenchyma in the same time frame, without reaching statistically significant difference with respect to the saline group (Fig 2D). On the other hand, the group receiving 6 μg BLM had a significantly higher hypo-aerated tissue already at day 7 and lasted at all the observed time-points (p<0.01), besides showing a marked increase in non-aerated areas at day 21 (p<0.05) (Fig 2E).

As expected, the saline group had 77–80% normo-aerated tissue with 20–23% hypo-aerated tissue throughout the study (S1B Fig).

#### Functional parameters

**Fig 3:**
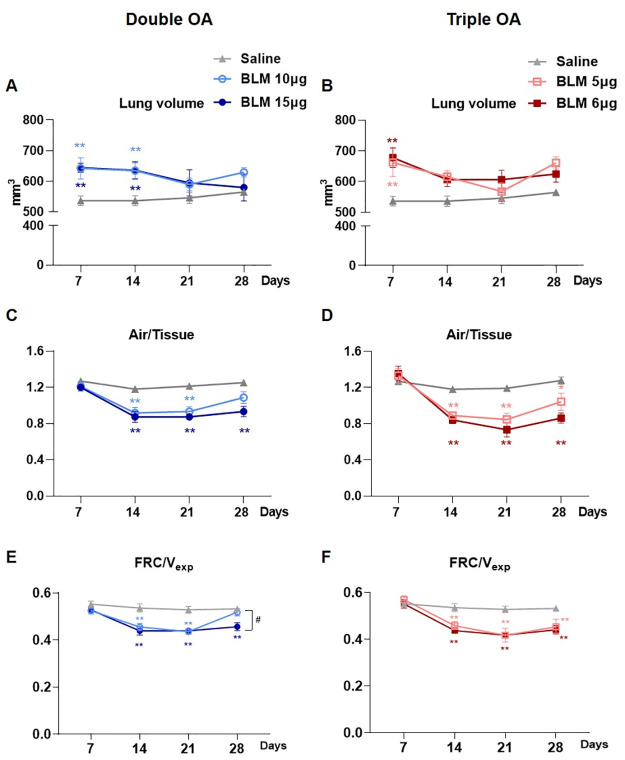
Longitudinal lung function readouts. Longitudinal analyses of the lung volume detected by micro-CT in mice treated with double (A) or triple (B) doses of BLM, compared with the saline group. Longitudinal quantification of air/tissue ratio in double (C) or triple (D) doses of BLM. Longitudinal quantification of FRC/V_exp_ in double (E) or triple (F) doses of BLM. Data are shown as mean ± SEM. The statistical differences were calculated by two-way ANOVA followed by Tukey’s test (*p<0.05; **p<0.01 vs. saline group. ## p<0.01 comparing 10 μg vs. 15 μg BLM). N = 5 per group.

Animals treated with a double dose of either 10 or 15 μg BLM had a significantly higher (p<0.01) lung volume with respect to saline at days 7 and 14, whilst no differences between groups were seen at days 21 and 28 (Fig 3A). On the other hand, mice receiving the triple OA of BLM at both doses had a prominent increase of lung volume at day 7 (p<0.01), whereas from day 14 no significant differences were revealed compared to saline (Fig 3B).

As regards the air/tissue ratio, at day 7 no differences between the groups receiving double BLM and saline were revealed. However, a markedly lower ratio (p<0.01) was observed in mice receiving either 10 or 15 μg BLM at days 14 and 21. Between days 21 and 28, the air/tissue ratio slightly increased in the 10 μg group but was not significantly different than in saline at day 28. In the 15 μg BLM group the ratio stabilized from 21 to 28 days, being still considerably lower with respect to saline (p<0.01) at the final time-point (Fig 3C). In the groups subjected to triple OA no differences were detected at day 7 in the air/tissue (Fig 3D). The ratio significantly decreased in fibrotic mice dosed with either 5 or 6 μg from 14 to 21 days (p<0.01). Although a slight increase at 28 days was observed, the parameter remained significantly lower compared to saline (p<0.05 for 5 μg and p<0.01 for 6 μg) (Fig 3D).

Fig 3E and 3F show FRC normalized on total lung volumes. FRC/V_exp_ was not affected by either double nor triple BLM administration at 7 days. However, from day 14 to 21 its significant decline was observed in all BLM-treated groups compared to saline mice (p<0.01). At day 28, FRC/V_exp_ remained significantly lower compared to saline (p<0.01) in all BLM-treated groups, except for the group receiving the double dose of 10 μg BLM which reinstated (Fig 3E).

As expected, the total lung volume and functional respiratory biomarkers were constant for the saline group throughout the study.

### Histology

**Fig 4:**
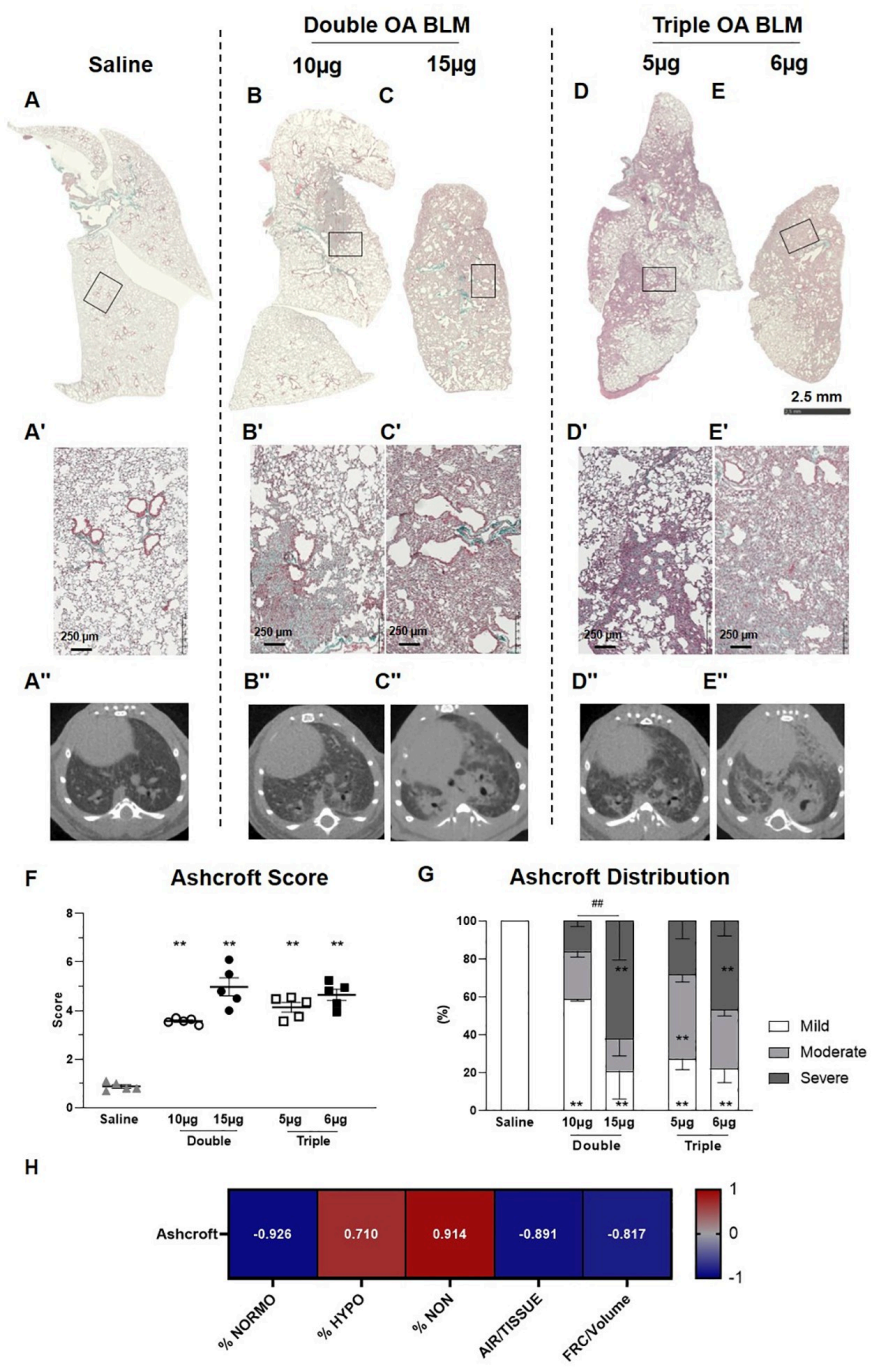
Histological staining. Representative micro-photographs of saline (A), BLM-double treated groups (B, C) and BLM-triple treated groups (D, E) stained with Masson’s trichrome at day 28. The squares indicate the selected zone with higher magnification (A´-E´). Representative transversal micro-CT scans of saline (A’’), double BLM-treated groups (B’’, C’’) and triple BLM-treated groups (D’’, E’’). (F) Ashcroft score for the saline and BLM groups. (G) Ashcroft frequency distribution in saline and BLM groups. (H) Spearman’s correlation between micro-CT parameters and Ashcroft Score. Data are shown as mean ± SEM. Statistical analysis was performed between the animal groups by one-way ANOVA followed by Tukey’s test (*p<0.05, **p<0.01 vs. saline; ## p<0.01 comparing low and high doses in double and triple OA groups, respectively). N = 5 per group.

Histological pictures of the whole lungs stained with MT of saline and BLM-treated groups are presented in Fig 4A–4E. As expected, histology at lower magnification revealed that both double (10, 15 μg) and triple (5, 6 μg) BLM treatment caused patchy fibrotic alterations of lung parenchyma characterized by fibroproliferative foci with various degrees of confluence at 28 days (Fig 4B–4E). On contrary, any parenchymal alteration was reported in the saline group (Fig 4A).

Micro-photographs at higher magnification showed that either double or triple administrations of BLM triggered mild to severe fibrosis, whereas the saline confirmed the normal morphology in this control group (Fig 4A’–4E’).

The corresponding transversal micro-CT scans of the mice reported in the above histology are shown in Fig 4A”–4E”.

The mean Ashcroft Score was significantly increased (p<0.01) in all groups receiving BLM compared to the saline group (Fig 4F). No statistically significant differences were revealed between BLM-treated groups. To better reflect the morphological alteration of lung parenchyma, the Ashcroft score was reported as the frequency distribution of mild (0–3), moderate (4), and severe (≥5) fibrotic lesions (Fig 4G) [10]. The double (10 μg) BLM treatment induced moderate to severe fibrosis with respect to the saline group, but within a limited area of parenchyma (Fig 4B). On the other hand, in all the other groups treated with BLM a more distributed injury in lung parenchyma was evident (Fig 4C–4E), with presence of both moderate and severe fibrosis. In particular, the highest % severe fibrosis was observed in mice receiving the double dose of 15 μg (62±20%) and the triple dose of 6 μg (47±8%). Between the two groups receiving a double OA of BLM a significant difference in terms of Ashcroft frequency distribution was revealed (p<0.01).

Spearman correlations between micro-CT readouts and Ashcroft score were performed (Fig 4H). Positive and negative significant correlations with high Spearman coefficients were obtained (p< 0.05) for all CT readouts, except for lung volume (Spearman coefficient = 0.007, p>0.05, data not shown).

### BAL cell counts

**Fig 5:**
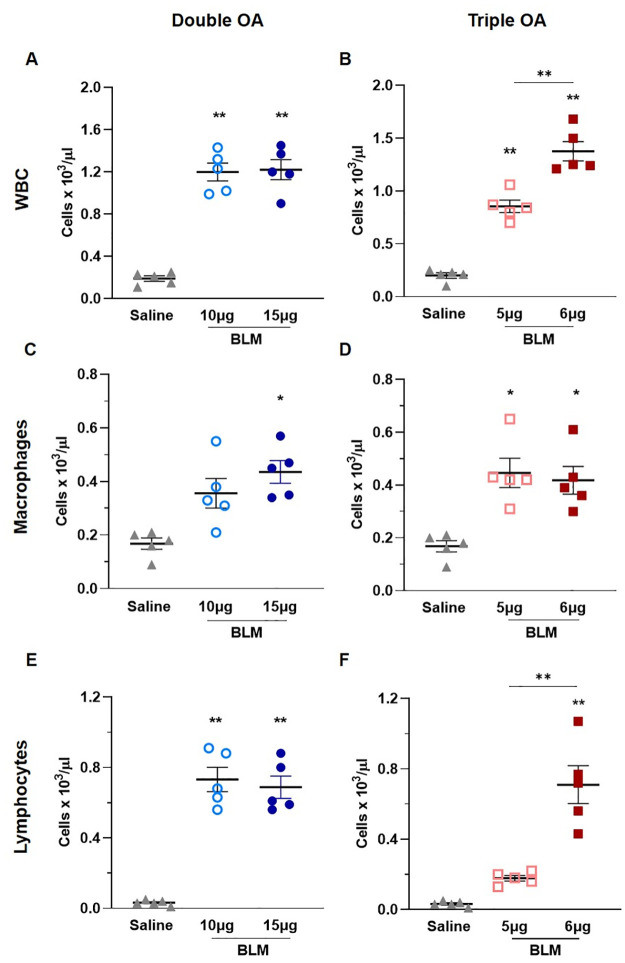
Inflammatory cells infiltration in BALF. Levels of total white blood cells (A, B), macrophages (C, D) and lymphocytes (E, F) in BALF of animals receiving saline, double and triple doses of BLM. Data are shown as mean ± SEM. Statistical significance was calculated between the animal groups by one-way ANOVA followed by Tukey’s test (*p<0.05; **p<0.01). N = 5 per group.

At day 28, after animals’ euthanasia, the BALF was harvested and leukocytes were counted. As expected, the total WBC were significantly increased in all the groups receiving BLM, compared with saline (p<0.01) (Fig 5A and 5B). A significant difference was revealed between the two groups treated with the triple BLM dose (Fig 5B), as the WBC count was much higher following administration of 6 μg (p<0.01).

As concerns leukocyte subpopulations, macrophages were markedly higher in the group receiving the double dose of 15 μg BLM compared to saline (p<0.05), whereas the double dose of 10 μg evoked a moderate, non-significant increase (Fig 5C). On the other hand, triple OA treatment with BLM induced a prominent increase in macrophages (p<0.05) at both doses compared to the saline group (Fig 5D). Following the double administration of BLM, lymphocytes were significantly increased compared to the saline group (p<0.05) (Fig 5E). On the other hand, the triple dose of 5 μg BLM slightly increased lymphocytes, although not significantly, versus saline, whereas in mice receiving the triple dose of 6 μg markedly higher levels of lymphocytes were observed (p<0.01) as compared with either the saline or the 5 μg BLM groups (Fig 5F).

**Fig 6:**
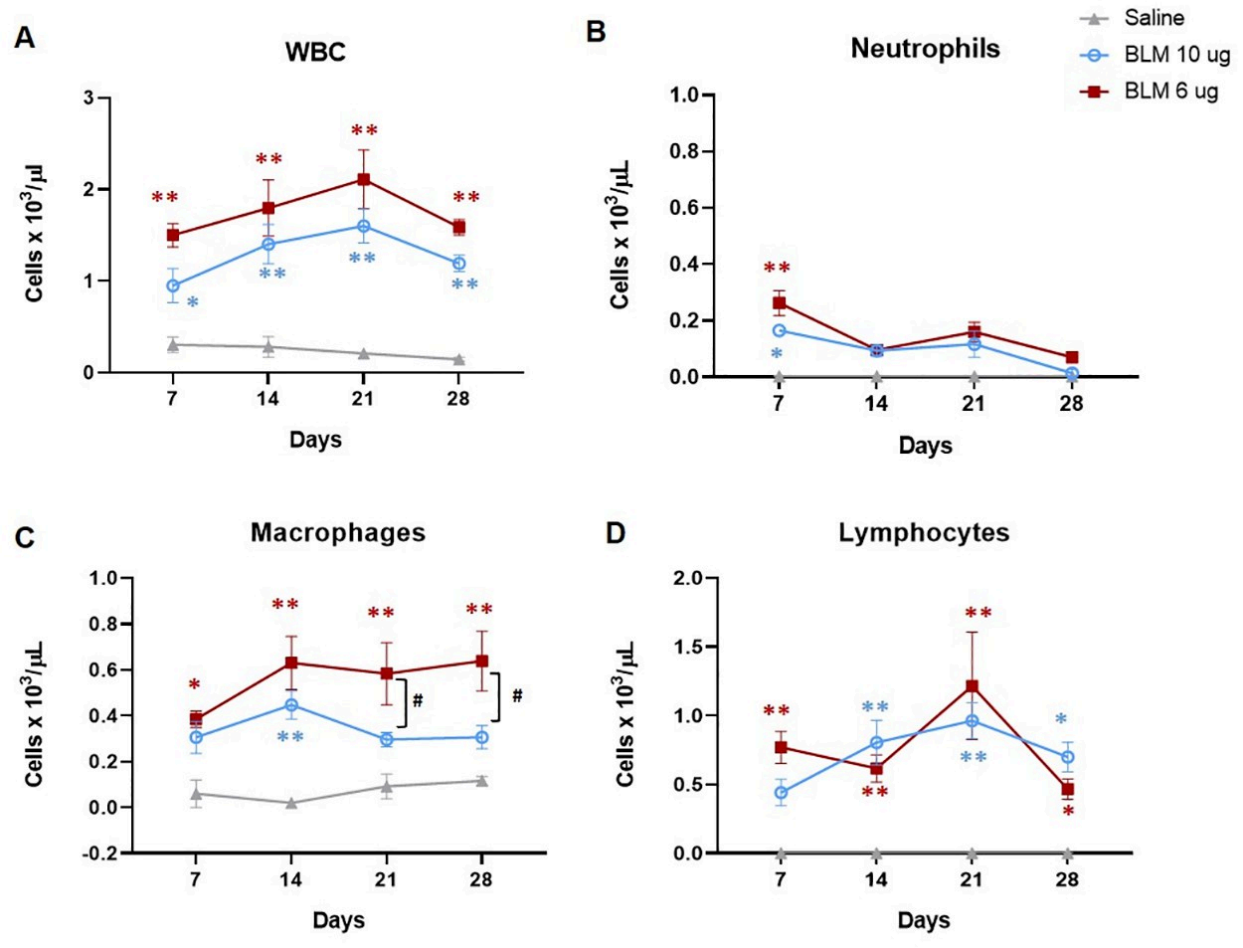
BAL cells count at different time-points. Levels of total white blood cells (A), neutrophils (B), macrophages (C) and lymphocytes (D) in BALF of animals receiving saline, the double dose of 10 μg and the triple dose of 6 μg at 7, 14, 21 and 28 days. Data are shown as mean ± SEM. The statistical differences were calculated by two-way ANOVA followed by Tukey’s test (*p<0.05; ** p<0.01 vs. saline; **#** p<0.05 BLM 6 μg vs. BLM 10 μg). N = 5 per group per time point.

Double (10 μg) and triple (6 μg) BLM administration were selected to further investigate the kinetic of the lung inflammation comparing the number of leukocytes in a time course experiment (Fig 6).

Even though total WBC in either BLM groups were significantly higher than saline across the study, their levels were slightly higher in mice receiving the triple BLM dose with respect to those treated with the double OA. However, no significant differences were revealed among the two BLM groups at any time-point (Fig 6A). Moreover, the levels of neutrophils, markers of inflammation, were comparable between the two groups across the study, being significantly higher than saline only at day 7 (Fig 6B). Instead, macrophages were higher in the group receiving the triple OA than in mice treted with the double BLM dose at the latter time points, and a significant difference (p<0.05) was revealed at day 21 and 28 (Fig 6C). Finally, the levels of lymphocytes were similar among the two BLM groups across the time course (Fig 6D).

## Discussion

Animal models play a crucial role either in understanding the pathobiology of fibrosis or in drug discovery. Animals of different species, gender and age, as well as different BLM dose regimens have been used to induce lung fibrosis [6, 8, 15, 20, 21].

In this study, BLM dosages and the administration scheme were readjusted in male C57Bl/6 mice, as they have been reported to be more susceptible to BLM-induced fibrosis with respect to female mice [8, 10].

Thus, we tested lower BLM doses compared to those used in female mice [6] applying two different administration schemes: a double OA (10, 15 μg each) and a triple OA (5, 6 and 7.5 μg each).

We hypothesized lower multiple doses of BLM might decrease acute toxicity and trigger sustained lung fibrosis up to 28 days, thus enabling an extended time window of 3 weeks (day 7 to 28) for pharmacological intervention, guaranteeing animal welfare throughout the experiment.

Micro-CT was performed longitudinally and the data were integrated with ex-vivo outcomes to select the best BLM regimen. Longitudinal imaging drastically reduced the number of mice per group and the intra-experimental variability, since the progression of the disease could be monitored in the same subjects [12, 13, 22, 23]. All the BLM-treated groups showed decreased normo- and increased hypo-aerated tissue; however, at day 7, a significant difference with respect to the saline group in hypo-aerated tissue was revealed only in mice dosed three times with 6 μg BLM (Fig 2E).

Moreover, non-aerated regions, reflecting the most severe fibrotic lesions, were found to be dose-dependently related to BLM. This was highlighted in mice treated twice with the highest BLM dose, in contrast with those receiving the double OA at 10 μg, in which a moderate fibrosis was detected. In order to assess lung fibrosis, besides considering the degrees of lung aeration, other micro-CT parameters, such as the total lung volume, the air/tissue ratio and the FRC normalized on total lung volume were proposed.

The increase of total lung volume in all BLM-treated mice at day 7 could be provoked by acute lung inflammatory phase, associated with fluid accumulation (edema) in the lungs [24]. Overall, this increase is not related to alterations in the proportions of air nor tissue volumes, as evidenced by the air/tissue ratio parameter (Fig 3C and 3D). However, at earlier time-points multiple processes take place: a balance between compensatory mechanisms, driven by inflammation, and collagen deposition, due to the initial stage of fibrosis, leads to an unaltered air/tissue ratio at 7 days, precluding its use to discriminate BLM group from saline. However, at later time-points it might be a useful parameter to describe lung fibrosis progression, as well as FRC/V_exp_ which significantly declined at the fibrotic stage. We believe that all the micro-CT parameters need to be considered when evaluating either disease development or response to anti-fibrotic drugs.

In agreement with the micro-CT data, histological analyses, depicted as Ashcroft score and its frequency distribution, revealed that all the BLM groups developed a sustained fibrosis ranging from 3.5–5.

However, the Ashcroft distribution marked a dose-dependent response to BLM, as only the higher doses either in double or triple regimen, increased significantly the percentage of severely injured areas, in line with non-aerated tissue detected by micro-CT. Moreover, we found highly significant Spearman coefficients correlating Ashcroft score with all CT parameters.

A dose-dependency was also revealed in inflammatory BAL cell counts, among the groups that underwent to the triple dose regimen, as the group receiving 6 μg had more prominent leukocytes infiltrate, driven mainly by lymphocytes. These data confirmed that when splitting the amount of BLM in three administrations, even slightly different amounts of BLM could result in a markedly different outcome after 28 days.

To further elucidate the kinetics of inflammation and fibrosis in the model we evaluated BAL cells populations at different time points in mice receiving either the double (10 μg) or triple (6 μg) BLM dose. We showed that the variations in BLM dose and regimen produced modest effects on the inflammatory phase, as no significant differences in BAL cells were revealed at day 7. Moreover, macrophages were higher in animals receiving the triple OA at later time points, indicating a key role for this cell type as a driver of fibrosis, as already reported in the classical BLM model [25].

Based on our findings, the higher doses applied with both protocols were able to trigger a sustained fibrosis up to 28 days.

However, although the double 15 μg BLM dose induced moderate body weight loss, histological quantification revealed a total Ashcroft score close to 5 with predominant severe fibrotic lesions. These findings, along with micro-CT outcomes, revealed a significant increase in non-aerated areas as well as a marked decrease in air/tissue ratio, which led us to consider this protocol as too severe. On the other hand, the triple administration corroborated our hypothesis: both 5 and 6 μg doses were able to induce relevant changes in lung fibrosis markers detected by micro-CT and histology, without causing excessive distress and body weight loss in animals.

In drug discovery, the balance between the severity of the disease model and animal welfare is crucial, as pharmacological studies require animal manipulation once or twice daily, which may cause further stress. Moreover, the evaluation of the anti-fibrotic activity of novel compounds would require a sustained, but not excessively severe BLM-induced fibrosis. However, whether the lung fibrosis elicited in this model by the triple OA of 6 μg BLM can be modulated by a reference compound remains to be evaluated and will be addressed in future studies. We believe that a disease model triggering a sustained pulmonary fibrosis until day 28, allowing three weeks of pharmacological intervention, could be very useful to evaluate compounds for primary drug screening.

## Conclusions

In this study, we showed the pivotal role of micro-CT in developing an optimized mouse model of lung fibrosis. Micro-CT technology is increasingly being recognized as a fundamental translational tool in preclinical studies, as it allows investigators to get relevant three-dimensional information about disease progression as well as to detect regional differences within the lung parenchyma. Furthermore, longitudinal measurements can be carried out from each subject in a non-invasive manner, thus enabling a significant refinement of animal studies as well as a reduction in sample size.

Overall, this will permit a considerable decrease in costs and time to develop disease models. Moreover, the quantification of normo-, hypo- and non-aerated areas and functional parameters such as air/tissue ratio and FRC/V_exp_, provides a wide and detailed overview of the disease progression. These parameters represent useful tools to select the optimal protocol for BLM studies to be used for primary drug screening, corroborated by ex-vivo measurements.

The continuous refinement of animal models and the integration with in-vivo imaging techniques (e.g. micro-CT and PET/CT) might be helpful within the drug discovery process, in order to increase the reliability of preclinical data and facilitate drug candidates’ translation into the clinic. Even though PET/CT can’t be used for primary screening due to its cost and complexity, this imaging technique could be extremely useful to better profiling the most promising anti-fibrotic drugs before clinical applications, as it enables to investigate the capacity of a drug to modulate the expression of relevant disease biomarkers by using specific probes [26].

However, as concerns secondary screening, different disease models are more suitable [22, 23], while we intended to use the disease model described herein for primary screening.

Furthermore, ventilatory parameters such as forced vital capacity (FVC), forced expiratory volume in the first 0.1 second (FEV_0.1_) and compliance of respiratory system (C_rs_) [27] provide relevant information about the lung function; however, the longitudinal application of this method on a large number of animals is quite challenging since mice need to be intubated and data could be affected by technical procedure.

Based on our findings, we identified a triple dose of 6 μg BLM as the optimal regimen to induce a sustained lung fibrosis that doesn’t spontaneously revert after 21 days, thus extending the time window for pharmacological treatment from 2 to 3 weeks.

## Supporting information

S1 Fig Longitudinal micro-CT results in saline and triple OA of 7.5 μg BLM group. (A) Representative coronal micro-CT lung slices and 3D renderings at the end of expiration phase of saline at 7, 14, 21 and 28 days. (B) Lung aeration degrees expressed as percentage of normo-, hypo- and non-aerated tissues detected at 7, 14, 21, 28 days for the saline group. (C) Representative coronal micro-CT lung slices and 3D renderings at the end of expiration phase of triple OA of 7.5 μg BLM group at 7 and 14 days. (D) Lung aeration degrees expressed as percentage of normo-, hypo- and non-aerated tissues detected at 7 and 14 days for the triple OA of 7.5 μg BLM group. (E) Longitudinal quantification of air/tissue ratio in triple OA of 7.5 μg BLM. Data are shown as mean ± SEM. The statistical differences were calculated by two-way ANOVA followed by Tukey’s test (**p<0.01 vs. saline). N = 5 per group.
(TIF)
