# Supplementary figures and images for: Pivotal role of micro-CT technology in setting up an optimized lung fibrosis mouse model for drug screening
Source: PLoS One. 2022 Jun 15;17(6):e0270005. doi: 10.1371/journal.pone.0270005 (PMC9200302; doi:10.1371/journal.pone.0270005)

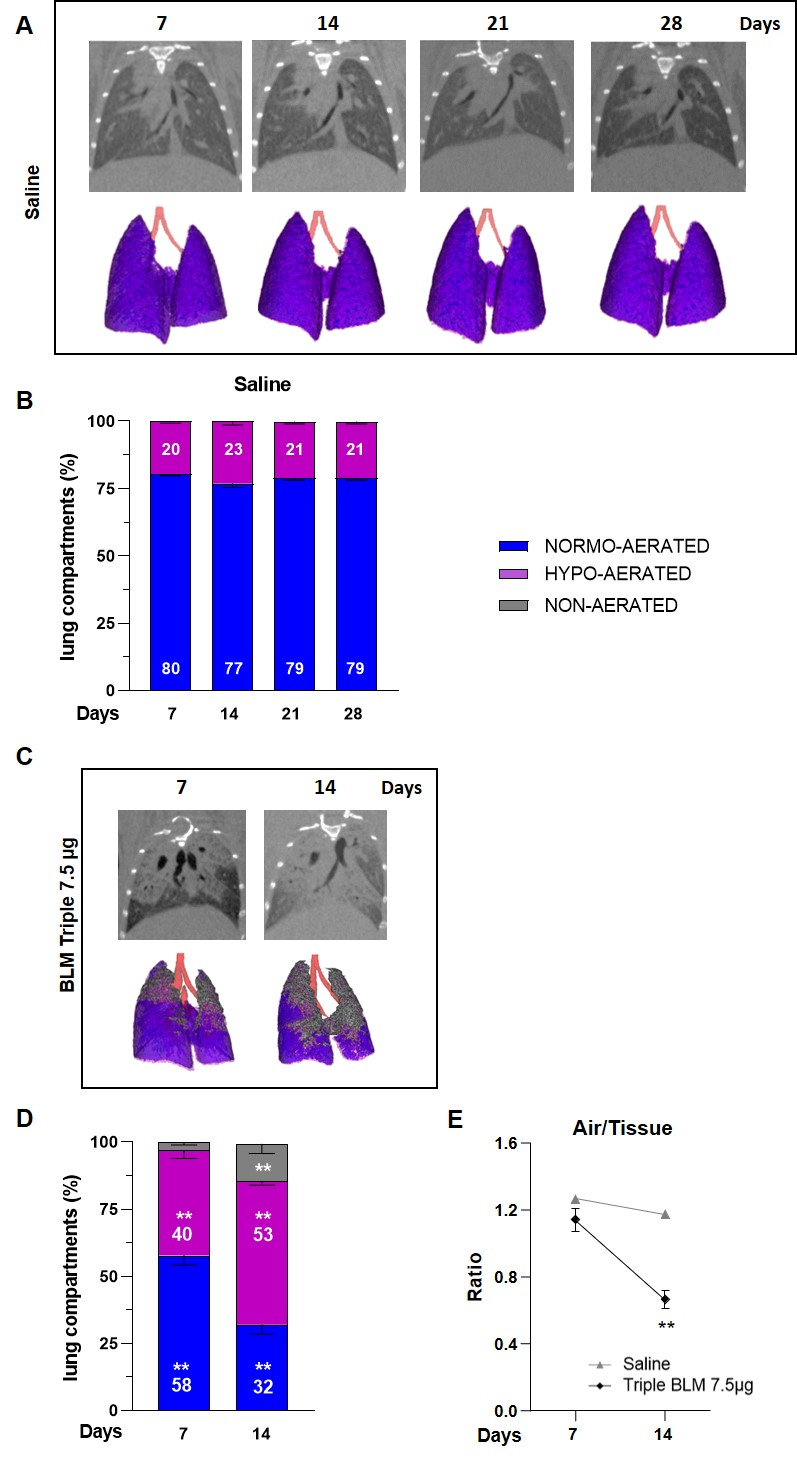

Supplement: S1 Fig — (A) Representative coronal micro-CT lung slices and 3D renderings at the end of expiration phase of saline at 7, 14, 21 and 28 days. (B) Lung aeration degrees expressed as percentage of normo-, hypo- and non-aerated tissues detected at 7, 14, 21, 28 days for the saline group. (C) Representative coronal micro-CT lung slices and 3D renderings at the end of expiration phase of triple OA of 7.5 μg BLM group at 7 and 14 days. (D) Lung aeration degrees expressed as percentage of normo-, hypo- and non-aerated tissues detected at 7 and 14 days for the triple OA of 7.5 μg BLM group. (E) Longitudinal quantification of air/tissue ratio in triple OA of 7.5 μg BLM. Data are shown as mean ± SEM. The statistical differences were calculated by two-way ANOVA followed by Tukey’s test (**p<0.01 vs. saline). N = 5 per group. (TIF) [file pone.0270005.s001.tif]
